# The Relationship Between Resilience and Mental Health in Chinese College Students: A Longitudinal Cross-Lagged Analysis

**DOI:** 10.3389/fpsyg.2020.00108

**Published:** 2020-02-05

**Authors:** Yin Wu, Zhi-qin Sang, Xiao-Chi Zhang, Jürgen Margraf

**Affiliations:** ^1^Department of Psychology, School of Social and Behavioral Sciences, Nanjing University, Nanjing, China; ^2^Students Affairs Department, Mental Health Education and Counseling Center, Nanjing University of Finance and Economics, Nanjing, China; ^3^Department of Clinical Psychology and Psychotherapy, Mental Health Research and Treatment Center, Ruhr-Universität Bochum, Bochum, Germany

**Keywords:** mental ill-being, positive mental health, resilience, cross-lagged analysis, college students

## Abstract

The relationship between resilience and mental health was examined in three phases over 4 years in a sample of 314 college students in China. The present study aimed to gain insight into the reciprocal relationship of higher levels of resilience predicting lower levels of mental ill-being, and higher levels of positive mental health, and vice versa, and track changes in both resilience, mental ill-being and positive mental health over 4 years. We used the Depression Anxiety Stress, the Positive Mental Health, and the Resilience Scales. Results revealed that first-year students and senior year students experienced higher negative mental health levels and lower positive mental health levels than junior year students. Cross-lagged structural equation modeling analyses showed that resilience could significantly predict mental health status in the short term, namely within 1 year from junior to senior year. However, the predicting function of resilience for mental health is not significant in the long term, namely within 2 years from freshman to junior year. Additionally, the significant predicting function of individuals’ mental health for resilience is fully verified for both the short and long term. These results indicate that college mental health education and interventions could be tailored based on students’ year in college.

## Introduction

Resilience refers to the process of adapting well in the face of adversity, trauma, tragedy, threats, or even significant sources of stress (American Psychological Association, 2014). According to many empirical studies, resilience is negatively correlated with indicators of mental ill-being, such as depression, anxiety, and negative emotions, and positively correlated with positive indicators of mental health, such as life satisfaction, subjective well-being, and positive emotions (Hu et al., 2015).

Some studies have shown that resilience is negatively correlated with depression and anxiety (Miller and Chandler, 2002; Nrugham et al., 2010; Wells et al., 2012; Poole et al., 2017; Shapero et al., 2019). Skrove et al. (2012) found that resilience characteristics are associated with lower anxiety and depression symptom levels. Anyan and Hjemdal (2016) indicated that resilience partially mediated the relationship between stress, and symptoms of anxiety, and depression. Goldstein et al. (2013) argued thatinternal resilience is both a compensatory and protective factor for depression symptoms in the context of sexual abuse among emerging adults transitioning out of child welfare. Poole et al. (2017) pointed out that resilience independently predicted symptoms of depression and moderated the association between adverse childhood experiences and depression. Shapero et al. (2019) determined that resilience significantly moderated the relationship between emotional reactivity and depressive symptoms. Anderson (2012) demonstrated that, among all aspects of resilience, the equanimity and meaning factors are most related to depression. Building resilience may be one way of preventing adolescent depression.

In addition, resilience showed significant correlation with positive mental health indicators, such as life satisfaction and subjective well-being (Haddadi and Besharat, 2010; Vitale, 2015; Satici, 2016; Tomyn and Weinberg, 2016). Tomyn and Weinberg (2016) found a moderate, positive correlation between resilience and subject well-being. Satici (2016) showed that resilience positively predicts subjective well-being through the mediating role of hope. Abolghasemi and Varaniyab (2010) found that psychological resilience and perceived stress explained 31 and 49%, respectively, of the variance of life satisfaction based on multiple regression analysis. Vitale (2015) demonstrated that resilience contributed to the positive outcome of life satisfaction in young adults with a history of childhood trauma. Smith (2009) showed that resilience and positive emotions might have a reciprocal influence on each other.

The studies addressing the relationship between resilience and mental health are mostly cross-sectional studies, while data analysis methods are centered on correlation and regression analysis (Abolghasemi and Varaniyab, 2010; Anderson, 2012; Skrove et al., 2012; Goldstein et al., 2013; Vitale, 2015; Tomyn and Weinberg, 2016), with some studies using the intermediate effect or regulatory effect analysis (Liu et al., 2012; Satici, 2016; Ding et al., 2017; Poole et al., 2017; Shapero et al., 2019). However, follow-up studies are generally insufficient: the temporal effects relationship between resilience and mental health could not be determined.

Many studies focus on the predictive function of resilience for mental health indicators (Goldstein et al., 2013; Vitale, 2015; Satici, 2016). And correspondingly, most intervention studies pay attention to the influence of resilience training to the improvement of mental health status. For example, in a meta-analysis, Dray et al. (2017) found that resilience-focused interventions were effective relative to a control in reducing depressive and anxiety symptoms for children and adolescents, particularly if a cognitive-behavioral therapy based approach is used. Waugh and Koster (2015) revealed that there was evidence that positivity training interventions aimed at increasing well-being, positive emotions and resilience had beneficial effects on depression. There are few studies that assessed mental health’s influence on resilience. Regarding the impact of mental ill-being on resilience, Pollack et al. (2004) found that compared with the general population, individuals with anxiety disorders exhibit less resilience. In terms of the impact of positive mental health on resilience, Tugade et al. (2005) argued that positive emotions served an important function in the ability of resilient individuals to rebound from stressful encounters. Nevertheless, the bidirectional causality between both sides has not been clearly explored. Thus, the present study—as a follow-up study remedying the shortcomings of existing studies—examines the temporal effects between resilience and mental health status based on a cross-lagged analysis.

Furthermore, previous studies have concentrated on separately analyzing the relationship between resilience and mental ill-being indicators or positive indicators. Based on the double-factor model of mental health (Keyes, 2005; Suldo and Shaffer, 2008), the present study introduces both negative and positive indicators to the mental health evaluation system. On that basis, it further provides support for studying the correlation between resilience and mental health, to contribute to the improvement of college students’ resilience and mental health status.

Therefore, the present study aimed to gain insight into the reciprocal relationship of higher levels of resilience predicting lower levels of mental ill-being, and higher levels of positive mental health, and vice versa. The Resilience Scale (RS-11, Schumacher et al., 2005), Depression Anxiety Stress Scale (DASS-21, Lovibond, 1995), and Positive Mental Health Scale (PMHS, Lukat et al., 2016) were applied in a college student sample in China three time over 4-year periods. The RS-11 was used to assess resilience that is associated with healthy development and psychosocial stress-resistance (Schumacher et al., 2005). DASS was a measure that captured three aspects of mental ill-being, including depression, anxiety, and general stress (Lovibond, 1995). And the PMHS was applied as a valid short unidimensional measure of general emotional well-being (Lukat et al., 2016). Furthermore, the survey continued for 4 years in order to track changes in both resilience, mental ill-being and positive mental health overtime.

Based on earlier empirical evidence that resilience, mental ill-being and positive mental health were associated with each other cross-sectionally (Miller and Chandler, 2002; Haddadi and Besharat, 2010; Nrugham et al., 2010; Wells et al., 2012; Vitale, 2015; Satici, 2016; Tomyn and Weinberg, 2016; Poole et al., 2017; Shapero et al., 2019) and that resilience played a predictive function for mental health indicators (Goldstein et al., 2013; Vitale, 2015; Satici, 2016), we hypothesized that (1) resilience would negatively correlate with DASS score and positively correlate with PMHS score; (2) resilience at T1 would predict DASS at T2 and vice versa; resilience at T2 would predict DASS at T3 and vice versa; (3) resilience at T1 would predict PMHS at T2 and vice versa; resilience at T2 would predict PMHS at T3 and vice versa.

## Materials and Methods

### Participants and Procedures

This project was part of the Bochum Optimism and Mental Health (BOOM) research project (Schönfeld et al., 2016). All participants were students at Nanjing University, China. The study was approved by the Ethics Committee of the Faculty of Psychology of the Ruhr-Universität Bochum and the Academic Ethics Committee of Nanjing University.

Based on the convenience sampling method, participants were recruited at the baseline year in 2012. Surveys were administered on the same participants three times: September 2012 (T1), September 2014 (T2), and September 2015 (T3). With the first survey, the level of resilience, mental ill-being, and positive mental health were evaluated when the participants were college freshmen. With the second and third surveys, the same three variables were evaluated when the participants were college juniors and seniors. The instruments were applied with pencil and paper. The data were collect at the different occasions to explore the different relationships in an extended period (2 years between the first and second measurement wave) and a short period (1 year between the second and third). The voluntary and confidential nature of their involvement in this study was clearly communicated to all students involved. Participants gave their informed consent orally one by one before participation. Informed consent had to be given orally, as no written materials were exchanged. This consent procedure was approved by the Academic Ethics Committee of Nanjing University. Participants received a gift (approximately $1.50) after completing each survey.

There were overall 1,064, 695, and 497 college students participating in the surveys conducted at T1, T2, and T3, respectively. Participants who completed less than 80% of the three target scales, who were suspected not to respond sincerely (i.e., all the answers were the same), or who missed one or more surveys were excluded. Finally, answers from 314 participants were included for further statistical analyses. Of these participants, there were 167 men and 147 women. The average age (at T1) of the longitudinal sample was 18.23 ± 0.76, ranging from 17 to 21. According to the result of T-test analysis, no significant differences were found between participants validly responding at all time points (*n* = 314), and dropouts or the ones who did not respond validly at any time (*n* = 750) concerning gender, resilience, depression, anxiety, stress, or positive mental health at T1. And the effect sizes were 0.014, 0.049, −0.054, −0.003, −0.017, and 0.034 respectively. According to G*Power, in order to have a power of 0.80 at an alpha-level of 0.05, the effect size (*d*) need to be more than 0.17, with the two groups of 314 and 750 participants in the T-test analysis. But the effect size here is relatively low to achieve the required power.

### Measures

#### Resilience Scale (RS-11)

The Resilience Scale (RS-25), as originally created by Wagnild and Young (1993), has been widely applied in many studies. Schumacher et al. (2005) created a German version of the scale, with fewer items (RS-11). A Chinese version of RS-11 was created through a translation and editing process by Gao et al. (2013). Resilience, in the shorter11-item version, is conceptualized as a protective personality factor that is associated with healthy development and psychosocial stress-resistance. The RS-11 is a unidimensional scale, using a 7-point Likert scale ranging from 1 (strongly disagree) to 7 (strongly agree). Higher total scores represent higher resilience levels. For our three surveys, the Cronbach’s *α* coefficient ranged from 0.83 to 0.87.The Compound Reliability coefficients ranged from 0.82 to 0.96. The Extracted Average Variance coefficients ranged from 0.56 to 0.67.The Omega McDonald’s coefficients ranged from 0.84 to 0.88.

#### Depression Anxiety Stress Scale (DASS-21)

The Depression Anxiety Stress Scale (DASS) was originally created by Lovibond (1995) and was used for assessing symptoms of depression, anxiety, and stress as outcome variables of daily stressors. Henry and Crawford (2005) verified that DASS-21, the short version of DASS, was reliable and valid for ordinary populations. A simplified Chinese version of DASS-21 was created through a translation and editing process by Gong et al. (2010). DASS-21 is composed of three sub-scales, including the depression, anxiety, and stress scale. Each sub-scale has 7 items that use a 4-point Likert scale ranging from 0 (did not apply to me at all) to 3 (applied to me very much or most of the time). This study measures the level of mental ill-being by using DASS-21. For our three surveys, the Cronbach’s *α* coefficient ranged from 0.87 to 0.89, from 0.84 to 0.85, from 0.84 to 0.87, for the depression, anxiety, and stress sub-scale, respectively. The Compound Reliability coefficients ranged from 0.65 to 0.88, from 0.68 to 0.86, from 0.74 to 0.86, respectively. The Extracted Average Variance coefficients ranged from 0.51 to 0.65, from 0.57 to 0.62, from 0.58 to 0.64, respectively. The Omega McDonald’s coefficients ranged from 0.81 to 0.83, from 0.86 to 0.88, from 0.86 to 0.89, respectively.

#### Positive Mental Health Scale

The Positive Mental Health Scale (PMHS) was created by Trumpf et al. (2010) and consists of 14 items. The present study applies the short version revised by Lukat et al. (2016), which is composed of 9 items that use a 4-point Likert scale ranging from 0 (do not agree) to 3 (agree). A higher score represents a more healthy and positive mental health status. The scale assesses positive aspects of health and life experiences (e.g., I am often carefree and in good spirits, I enjoy my life, I manage well to fulfill my needs, I am in good physical and emotional condition). Lukat et al. (2016) showed that the short version is a unidimensional scale, with good reliability and validity. Before our study, there was no Chinese version of this scale. Thus, we applied the “Translation-Backtranslation-Revision” method to create the Chinese version for our study. All participating personnel for translating and back-translating are experts in German and Chinese. The Cronbach’s *α* coefficient ranged from 0.81 to 0.92 over the three surveys. The Compound Reliability coefficients ranged from 0.92 to 0.96. The Extracted Average Variance coefficients ranged from 0.60 to 0.69. The Omega McDonald’s coefficients ranged from 0.89 to 0.92.

### Statistical Analysis

SPSS24.0 (IBM Corp, 2010) was used to calculate the descriptive statistics and correlations between resilience and mental health status (including DASS and PMHS). Then, the mean changes in resilience, DASS, and PMHS across the 3 years were tested *via* repeated-measures multivariate analysis of variance (MANOVA) using SPSS24.0. In addition, JASP (Wagenmakers, 2014) was applied to calculate the reliability coefficient Omega McDonalds. And G*Power 3.1.9.4 (Buchner et al., 2019) was used for the power analysis.

Scores on resilience, DASS, and PMHS at T1, T2, and T3 were included for modeling and examining intra-individual changes over time using a cross-lagged analysis. Amos22.0 (Arbuckle, 2013) was applied for the cross-lagged analysis. The cross-lagged panel model was used to analyze the interactions and reciprocal influences between resilience and mental health over time. Cross-lagged path coefficients (i.e., predictive associations) between T1 and T2 for resilience (measured by RS) and mental health status (indexed by DASS and PMHS) were included, as well as the path coefficients between T2 and T3. The cross-lagged paths denote to what extent the prior scores of one variable relate to subsequent scores of the other variable. The panel model further included stability coefficients between T1, T2, and T3 for RS, DASS, and PMHS to allow follow-up measurements (T2 and T3) to reflect residual change over time. We further included correlations (non-directional associations) between resilience and mental health status (including DASS and PMHS) at each of the three time-points (for T2 and T3, correlations were between residual errors). To test the model fit, we used the model testing indices including *χ*^2^/df, NFI, RFI, IFI, TLI, CFI, and RMSEA.

## Results

### Measurement Invariance Testing

As the present study is a repeated measure design, measurement invariance (MI) testing was performed to check if the constructs of the three scales were invariant over time.

#### Measurement Invariance of RS-11

**Figure 1:**
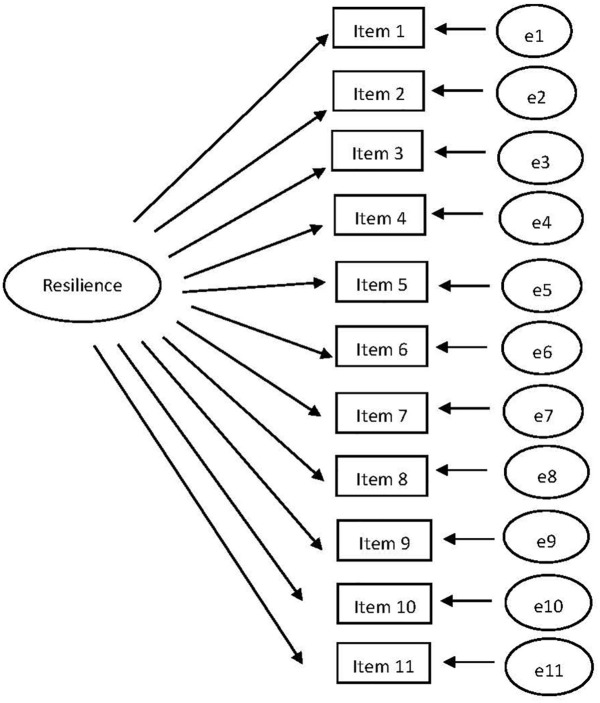
The CFA model with the unconstrained factor loadings and intercepts for RS-11.

The RS-11 is a unidimensional scale. The CFA model for RS-11 with the unconstrained factor loadings and intercepts is shown in Figure 1. Three CFA’s were conducted for T1, T2, and T3, separately. Next, we tested for measurement invariance, see Table 1 for the fit indices. CHIDIST (Δ*χ*^2^, Δdf) = 0.19. Δ*χ*^2^ is now insignificant, so invariance is established.

**Table 1:** The fit indices of unconstrained and measurement weight models for RS-11.

Model	*χ*^2^	df	CFI	TLI	RMSEA
Unconstrained	641.991	132	0.916	0.902	0.064
Measurement weights	667.276	152	0.914	0.908	0.060

#### Measurement Invariance of DASS-21

**Figure 2:**
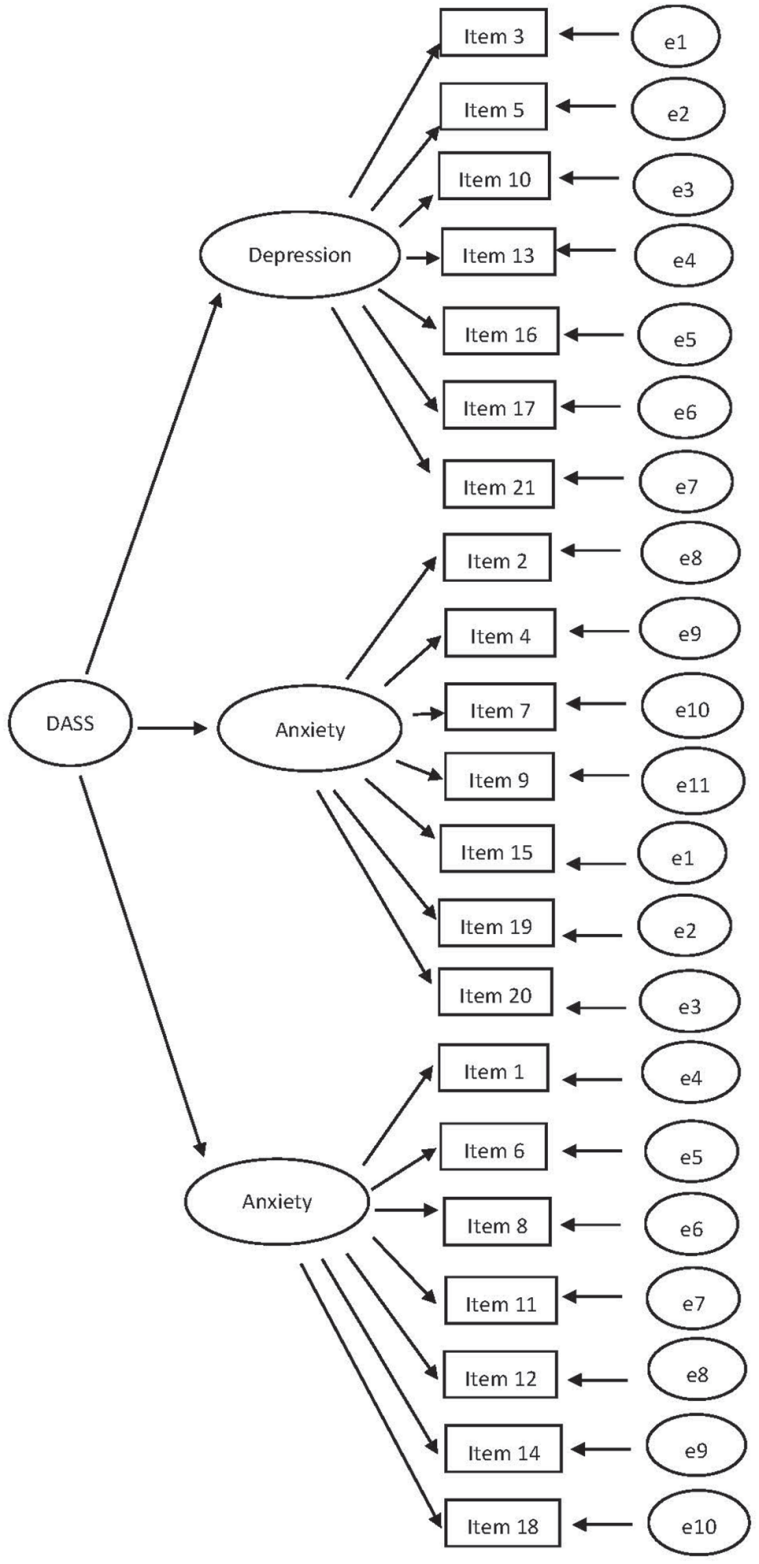
The CFA model with the unconstrained factor loadings and intercepts for DASS-21.

TheDASS-21 is a scale with three dimensions. The CFA model for DASS-21 with the unconstrained factor loadings and intercepts is shown in Figure 2. Three CFA’s were conducted for T1, T2, and T3, separately. Next, we tested for measurement invariance, see Table 2 for the fit indices. CHIDIST (Δ*χ*^2^, Δdf) = 0.06. Δ*χ*^2^ is now insignificant, so invariance is established.

**Table 2:** The fit indices of unconstrained and measurement weight models for DASS-21.

Model	*χ*^2^	df	CFI	TLI	RMSEA
Unconstrained	2599.91	558	0.894	0.868	0.062
Measurement weights	2649.63	594	0.853	0.856	0.060

#### Measurement Invariance of Positive Mental Health Scale

**Figure 3:**
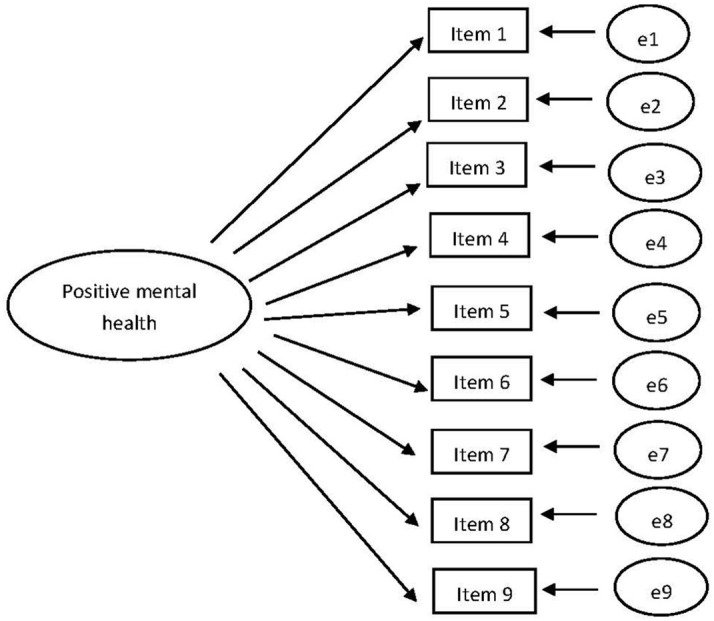
The CFA model with the unconstrained factor loadings and intercepts for PMHS.

The PMHS is a unidimensional scale. The CFA model for PMHS with the unconstrained factor loadings and intercepts is shown in Figure 3. Three CFA’s were conducted for T1, T2, and T3, separately. Next, we tested for measurement invariance, see Table 3 for the fit indices. CHIDIST (Δ*χ*^2^, Δdf) = 0.32. Δ*χ*^2^ is now insignificant, so invariance is established.

**Table 3:** The fit indices of unconstrained and measurement weight models for PMHS.

Model	*χ*^2^	df	CFI	TLI	RMSEA
Unconstrained	471.52	81	0.925	0.900	0.072
Measurement weights	489.505	97	0.925	0.916	0.066

### Relationship Between Resilience and Mental Health Status

As shown in Table 4, in the three surveys, the pairwise simultaneous and successive correlations between depression, anxiety, stress, and resilience were negative and significant, except with resilience in T1 and stress in T2.

**Table 4:** Means and standard deviations (SDs), as well as correlations among depression, anxiety, stress, and resilience at T1, T2, and T3.

	*M* ± SD	1	2	3	4	5	6	7	8	9	10	11	12
1. Depression (T1)	1.12 ± 1.49	1											
2. Depression (T2)	1.55 ± 2.92	0.15^**^	1										
3. Depression (T3)	2.24 ± 3.21	0.18^**^	0.42^**^	1									
4. Anxiety (T1)	2.47 ± 2.09	0.58^**^	0.13^*^	0.20^**^	1								
5. Anxiety (T2)	1.62 ± 2.09	0.11	0.84^**^	0.42^**^	0.18^**^	1							
6. Anxiety (T3)	2.46 ± 3.01	0.18^**^	0.34^**^	0.82^**^	0.32^**^	0.45^**^	1						
7. Stress (T1)	2.82 ± 2.76	0.50^**^	0.17^**^	0.21^**^	0.65^**^	0.17^**^	0.25^**^	1					
8. Stress (T2)	2.19 ± 3.12	0.15^**^	0.86^**^	0.39^**^	0.21^**^	0.82^**^	0.39^**^	0.23^**^	1				
9. Stress (T3)	3.01 ± 3.39	0.23^**^	0.34^**^	0.81^**^	0.31^**^	0.42^**^	0.83^**^	0.31^**^	0.45^**^	1			
10. Resilience (T1)	59.92 ± 7.30	−0.37^***^	−0.27^***^	−0.22^***^	−0.19^***^	−0.11^*^	−0.15^**^	−0.15^**^	−0.08	−0.14^*^	1		
11. Resilience (T2)	59.04 ± 8.44	−0.25^***^	−0.17^**^	−0.21^***^	−0.43^***^	−0.35^***^	−0.42^***^	−0.34^***^	−0.28^***^	−0.33^***^	0.44^***^	1	
12. Resilience (T3)	59.84 ± 8.26	−0.27^***^	−0.21^**^	−0.23^***^	−0.32^***^	−0.26^***^	−0.31^***^	−0.38^***^	−0.33^***^	−0.39^***^	0.37^***^	0.51^***^	1

*M, mean; SD, standard deviation; T1, the first survey; T2, the second survey; T3, the third survey*.

* p < 0.05;

** p < 0.01;

*** *p < 0.001*.

As shown in Table 5, the pairwise simultaneous and successive correlations were all positive and significant between resilience and positive mental health.

**Table 5:** Means and standard deviations (SDs), as well as correlations between positive mental health and resilience in T1, T2, and T3.

	*M* ± SD	1	2	3	4	5	6
1. Positive mental health (T1)	22.25 ± 4.21	1					
2. Positive mental health (T2)	30.35 ± 4.94	0.48^***^	1				
3. Positive mental health (T3)	29.11 ± 4.51	0.42^***^	0.60^***^	1			
4. Resilience (T1)	59.92 ± 7.30	0.60^***^	0.27^***^	0.28^***^	1		
5. Resilience (T2)	59.04 ± 8.44	0.45^***^	0.57^***^	0.45^***^	0.44^***^	1	
6. Resilience (T3)	59.84 ± 8.26	0.31^***^	0.41^***^	0.60^***^	0.37^***^	0.51^***^	1

*M, mean; SD, standard deviation; T1, the first survey; T2, the second survey; T3, the third survey*.

*** *p < 0.001*.

### Changes in Resilience and Mental Health Status Across Time

A repeated-measures MANOVA was conducted with time (T1, T2, and T3) as the within-group independent variable and the scores on resilience, depression, anxiety, stress, and PMHS as dependent variables. A significant effect of time was observed for depression [*F*(2, 314) = 18.08, *p* < 0.001, $\eta_{p}^{2}$ = 0.03], anxiety [*F*(2, 314) = 15.20, *p* < 0.001, $\eta_{p}^{2}$ = 0.02], stress [*F*(2, 314) = 9.54, *p* < 0.001, $\eta_{p}^{2}$ = 0.01], and PMHS [F(2, 314) = 506.84, *p* < 0.001, $\eta_{p}^{2}$ = 0.32]. The level of depression increased and reached a peak in T3.The level of anxiety and stress decreased first and then increased in a U-type tendency. The level of positive mental health increased first and then decreased in an inverted-U tendency. However, no significant effect of time was observed for resilience ($\eta_{p}^{2}$ = 0.003). According to G*Power, in order to have a power of 0.80 at an alpha-level of 0.05, the effect size (*f*) needs to be more than 0.10, with 314 participants in the MANOVA analysis. But the effect size here is relatively low to achieve the required power, except the effect of time for PMHS.

### Cross-Lagged Analysis for Resilience and Level of Mental Health

Based on the correlation analysis, by establishing a structural equation model and implementing a cross-lagged analysis for resilience and mental health level, the present study explored the bidirectional prediction relationship between resilience and negative mental health and between resilience and positive mental health.

**Figure 4:**
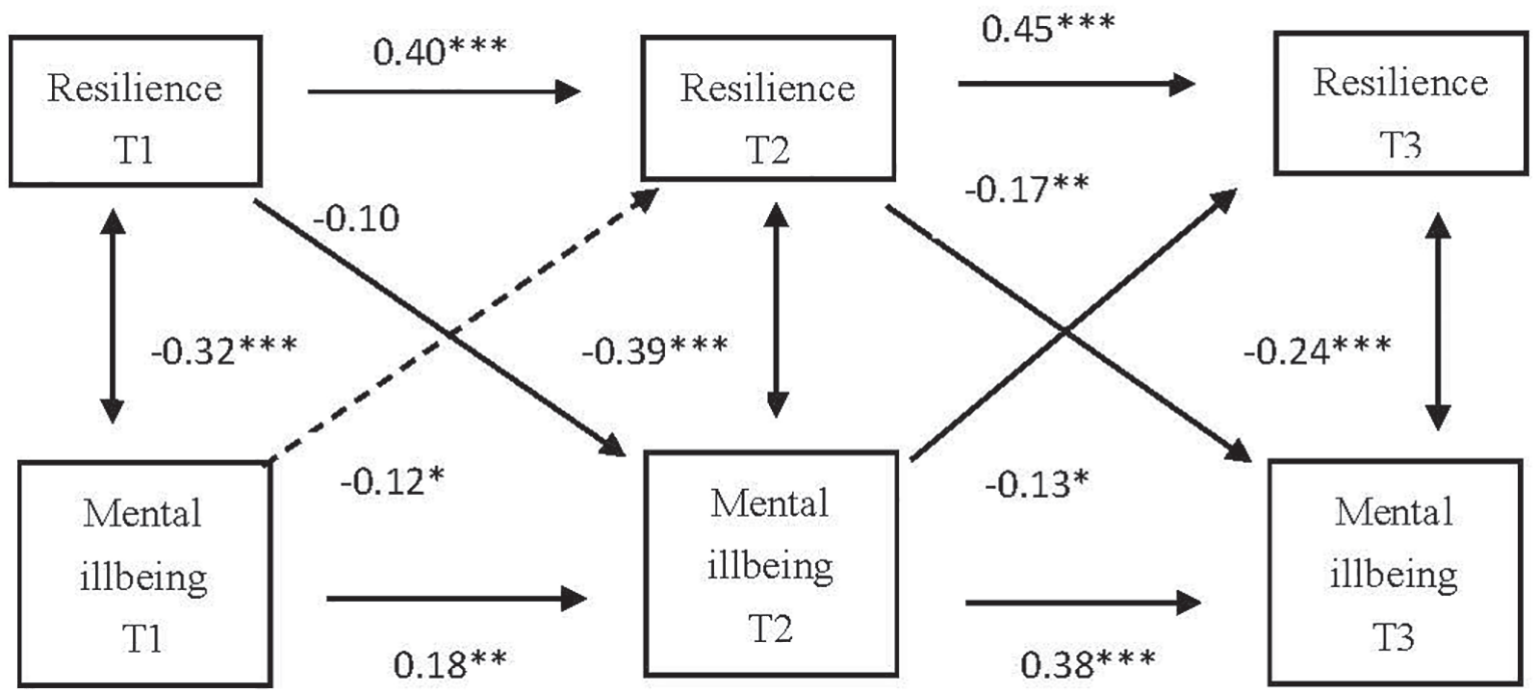
Cross-lagged analysis of the relationship between resilience and negative mental health. T1, the first survey; T2, the second survey; T3, the third survey; ^*^p < 0.05, ^**^p < 0.01, ^***^p < 0.001.

We explored the bidirectional prediction relationship between the variable resilience and mental ill-being. The overall fit of this initial measurement model was acceptable (*χ*^2^/df = 3.211, NFI = 0.919, RFI = 0.896, IFI = 0.928, TLI = 0.901, CFI = 0.926, and RMSEA = 0.083). As shown in Figure 4, the level of mental ill-being at T1 significantly and negatively predicted resilience at T2. The level of mental ill-being at T2 significantly and negatively predicted resilience at T3. Resilience at T2 significantly and negatively predicted the level of mental ill-being at T3.

**Figure 5:**
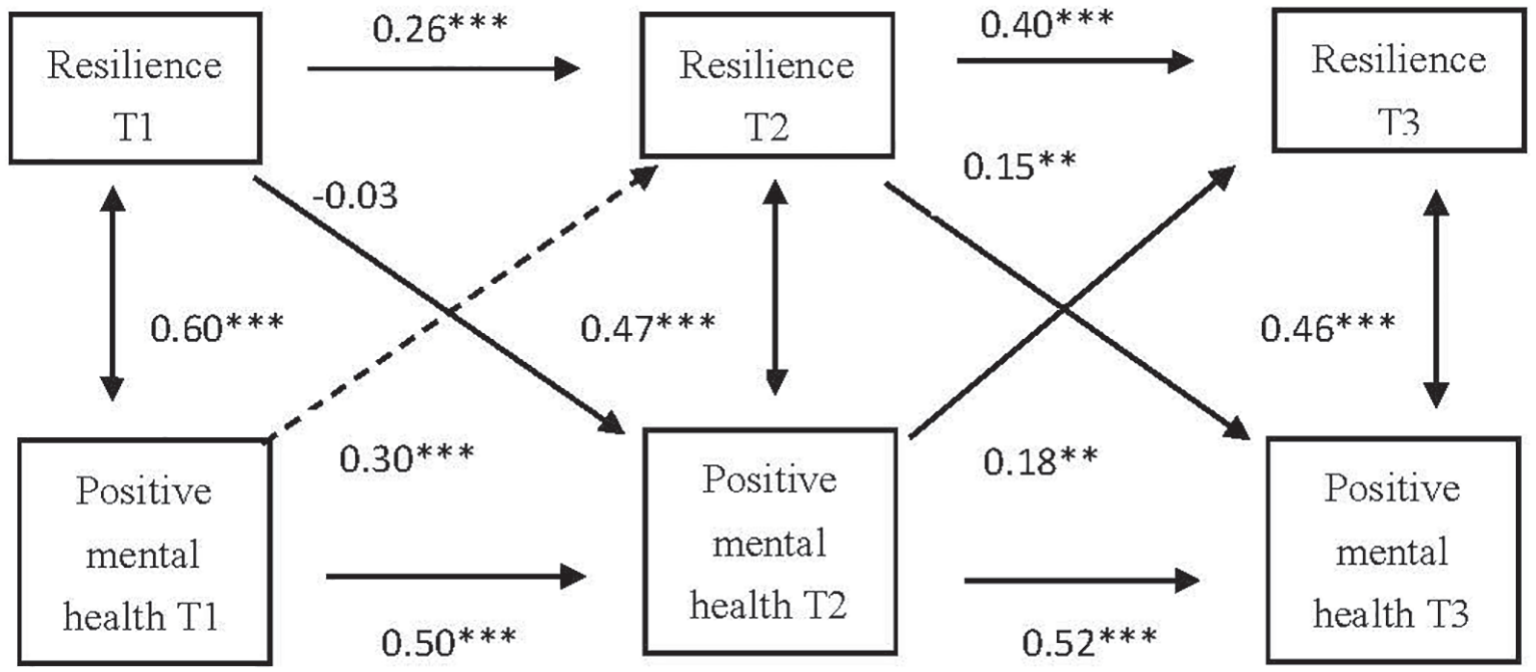
Cross-lagged analysis on the relationship between resilience and positive mental health. T1, the first survey; T2, the second survey; T3, the third survey; ^**^p < 0.01; ^***^p < 0.001.

Since positive mental health and resilience are both unidimensional variables, the bidirectional prediction relationship was explored between resilience and positive mental health, as two observed variables. The overall fit of this initial measurement model was acceptable (*χ*^2^/df = 4.228, NFI = 0.972, RFI = 0.893, IFI = 0.977, TLI = 0.912, CFI = 0.976, and RMSEA = 0.076). As shown in Figure 5, the level of positive mental health at T1 significantly and positively predicted resilience at T2. The level of positive mental health at T2 significantly and positively predicted resilience at T3. Resilience at T2 significantly and positively predicted the level of positive mental health at T3.

## Discussion

The present study is the first study to examine the bidirectional relationship between resilience and mental health status in three phases over 4 years using cross-lagged panel analysis in a college student sample. As expected, our analyses further revealed a significant reciprocal relationship between resilience and mental health status, indicating that resilience predicted the level of mental health status in short term of 1 year, and vice versa. And in the longer term of 2 years, mental health was found to predict resilience level. These findings broaden the cross-sectional results in earlier studies and extend an understanding of the relationship between resilience and mental health status in younger adults.

The present study is innovative in several ways. First, previous studies verified that resilience could predict mental health status (Goldstein et al., 2013; Vitale, 2015; Satici, 2016). Anderson (2012) indicated that administering screening measures such as the Resilience Scale was an efficient way to identify those students who may be at risk for depressive symptoms. However, according to the result of our study, this prediction was significant considering 1 year, but not 2 years. If the baseline and retest time interval is too long, the predictive effect of resilience on mental health is not significant. Meulen et al. (2018) pointed out that psychological resilience has a declining protective capacity for mental health disturbances over a medium time-span, specifically when corrected for baseline mental health disturbances.

Secondly, this study verified the significant influence of mental health level on resilience. While the majority of previous studies used mental health status only as an outcome measure, only a few studies have considered positive mental health as predictive factor (Pollack et al., 2004). Our empirical results add to this by showing that improved mental health is associated with increased resilience.

Thus, from the perspective of longitudinal development, the influence of resilience on mental health status has a chain effect: mental health status appears to influence resilience, while resilience further affects mental health status. As a result, individuals with lower baseline mental health levels and who encounter adversity later in their life should receive timely mental health education or intervention to enhance their level of resilience, coping capacity with adversity, and adaptability to the environment. This approach aims to prevent “the Matthew Effect,” which makes the strong stronger and the weak weaker. During this period, a preventive intervention could be offered for college students, to increase their autonomy, self-acceptance, environmental mastery, purpose in life, positive relations, and personal growth. Such interventions could reduce the risk of developing a mental disorder and experiencing other negative consequences by enhancing protective factors (Herrero et al., 2019). Oldfield et al. (2018) found that school connectedness may provide a role in promoting resilience for mental health for adolescents who were at risk due to poor parental attachment. So, it is beneficial to enhance positive relationship between students and teachers to increase students’ sense of belonging, which could improve their self-identity and social skills. Such education and interventions can support students as they adapt to different challenges and allow them to increase their mental health status in their studies and life.

Thirdly, this study included both mental ill-being and positive indicators of mental health, based on the double-factor model of mental health (Keyes, 2005; Suldo and Shaffer, 2008). Results showed a picture of how mental health status fluctuates in college students across time.

Regardingmental ill-being, the depression level was lower in freshmen, higher in juniors, and highest in seniors. The anxiety and stress level was higher in freshmen and seniors and lower in juniors. College students, when faced with environmental changes and transitional periods in life during freshman and senior years, may experience more negative emotions than during their more stable junior years. These findings are consistent with previous studies about the mental health status of college students in China (Xue, 2001; Li, 2003) and the United States (Soet and Sevig, 2006). When freshmen enter their university, they will face a change in the lives, such as new social relationships and contexts without the support of parents or long-time friends, academic pressure, stress during exams, and social disconnection. And this is considered a stress factor and a heightened risk for psychopathology (Herrero et al., 2019). Beiter et al. (2015) pointed that post-graduation plans is one of the top 10 sources of concern for college students, and finding a job after graduation is one of the first four concerns directly relate to college student life. The seniors appear to have increased levels of depression and anxiety because they will soon be on the job market and face the pressure of finding a job or passing post-graduate entrance examination. These results indicate that college mental health education and interventions could be tailored based on students’ year in college. It is necessary to reinforce mental health education for college students during their freshmen and senior years, especially by providing mental health education services for stress management and anxiety relief training for those students. In addition, college mental health educators need to pay attention to seniors’ higher levels of depression and strengthen screening for depressive symptoms for students at this stage. It is important to design programs to help freshman settle into college life, as well as to help seniors prepare for jobs or graduate school. In addition, maybe it is also beneficial to prepare juniors for what they will need to accomplish in their senior year and hopefully reduce their stress, anxiety, and depression then (Beiter et al., 2015).

In terms of positive mental health, the present study found that freshmen had a relatively lower level of positive mental health. As they grow older, college students appear to meet the challenges of their studies and life more confidently and steadfastly than before. Their level of positive mental health reached a peak in their junior year but then decreased in their senior year, possibly owing to the increase in stress in multiple areas. The relatively lower level of positive mental health in freshmen leaves room for mental health education. In other words, interventions should be implemented to enhance their positive mental health by recognizing and applying positivity; sensing and appreciating the experiences of positivity, training, and forming positive thinking; and establishing and maintaining positive interpersonal relationships (Duan and Bu, 2018).

There are some limitations to the present study. First, the large drop-out was a potential limitation of the research. There were 1,064 freshmen participants in the surveys conducted at T1. However, only 497 participants finished all three waves of the survey due to difficulties in following up the participants. Second, the result of power analysis for the T-test and MANOVA analysis was not ideal in present study, as the effect size was relatively low. The research design, especially the sampling process, needs to be improved in the future research. For example, participants could be recruited from different universities to reduce the overlap degree of the two population distribution and improve the effect size. Third, the surveys were applied with pencil and paper. However, an online registration would have been much safer, registering the time, guaranteeing a higher quality of the data and better risk of coding errors of the same ones. Four, the current study only investigated the reciprocal relationship between resilience and mental health status in Chinese college students. In other age periods or population groups, the causal direction need to be explored in the future research. Five, the present study is descriptive research, so the causal direction needs to be explored in future research. Consequently, the casual relationships need to be examined by intervention research in the future. Six, resilience is required in response to different adversities, ranging from ongoing daily hassles to major life events (Fletcher and Sarlcar, 2013). Increased opportunities for exposure to adversity and life experience may be an important factor affecting the relationship between trait resilience and mental health (Hu et al., 2015). So, future studies could recruit participants who experience adversity, such as lovelorn, family misfortune, academic difficulties, or employment difficulties, to explore the moderation effect of adversity on the relationship between resilience and mental health status.

## Conclusion

In sum, the current study provides preliminary evidence of a mutually reducing relationship between resilience and mental ill-being and the mutually enhancing relationship between resilience and positive mental health in the short term of 1 year. And the significant influence of mental health level on resilience was presented in the long term of 2 years. Future intervention research is warranted to further verify this reciprocal relationship.

## Author Contributions

YW designed the study, performed the statistical analyses, and drafted the manuscript. ZS and X-CZ organized the data collection. JM designed the project.

### Conflict of Interest

The authors declare that the research was conducted in the absence of any commercial or financial relationships that could be construed as a potential conflict of interest.
